# Deep Sequencing of ESTs from Nacreous and Prismatic Layer Producing Tissues and a Screen for Novel Shell Formation-Related Genes in the Pearl Oyster

**DOI:** 10.1371/journal.pone.0021238

**Published:** 2011-06-22

**Authors:** Shigeharu Kinoshita, Ning Wang, Haruka Inoue, Kaoru Maeyama, Kikuhiko Okamoto, Kiyohito Nagai, Hidehiro Kondo, Ikuo Hirono, Shuichi Asakawa, Shugo Watabe

**Affiliations:** 1 Department of Aquatic Bioscience, Graduate School of Agricultural and Life Sciences, The University of Tokyo, Bunkyo, Tokyo, Japan; 2 Mikimoto Pharmaceutical CO., LTD, Ise, Mie, Japan; 3 Pearl Research Institute, Mikimoto Co., Ltd, Shima, Mie, Japan; 4 Laboratory of Genome Science, Graduate School of Marine Science and Technology, Tokyo University of Marine Science and Technology, Minato, Tokyo, Japan; Biodiversity Insitute of Ontario - University of Guelph, Canada

## Abstract

**Background:**

Despite its economic importance, we have a limited understanding of the molecular mechanisms underlying shell formation in pearl oysters, wherein the calcium carbonate crystals, nacre and prism, are formed in a highly controlled manner. We constructed comprehensive expressed gene profiles in the shell-forming tissues of the pearl oyster *Pinctada fucata* and identified novel shell formation-related genes candidates.

**Principal Findings:**

We employed the GS FLX 454 system and constructed transcriptome data sets from pallial mantle and pearl sac, which form the nacreous layer, and from the mantle edge, which forms the prismatic layer in *P. fucata*. We sequenced 260477 reads and obtained 29682 unique sequences. We also screened novel nacreous and prismatic gene candidates by a combined analysis of sequence and expression data sets, and identified various genes encoding lectin, protease, protease inhibitors, lysine-rich matrix protein, and secreting calcium-binding proteins. We also examined the expression of known nacreous and prismatic genes in our EST library and identified novel isoforms with tissue-specific expressions.

**Conclusions:**

We constructed EST data sets from the nacre- and prism-producing tissues in *P. fucata* and found 29682 unique sequences containing novel gene candidates for nacreous and prismatic layer formation. This is the first report of deep sequencing of ESTs in the shell-forming tissues of *P. fucata* and our data provide a powerful tool for a comprehensive understanding of the molecular mechanisms of molluscan biomineralization.

## Introduction

**Figure 1:**
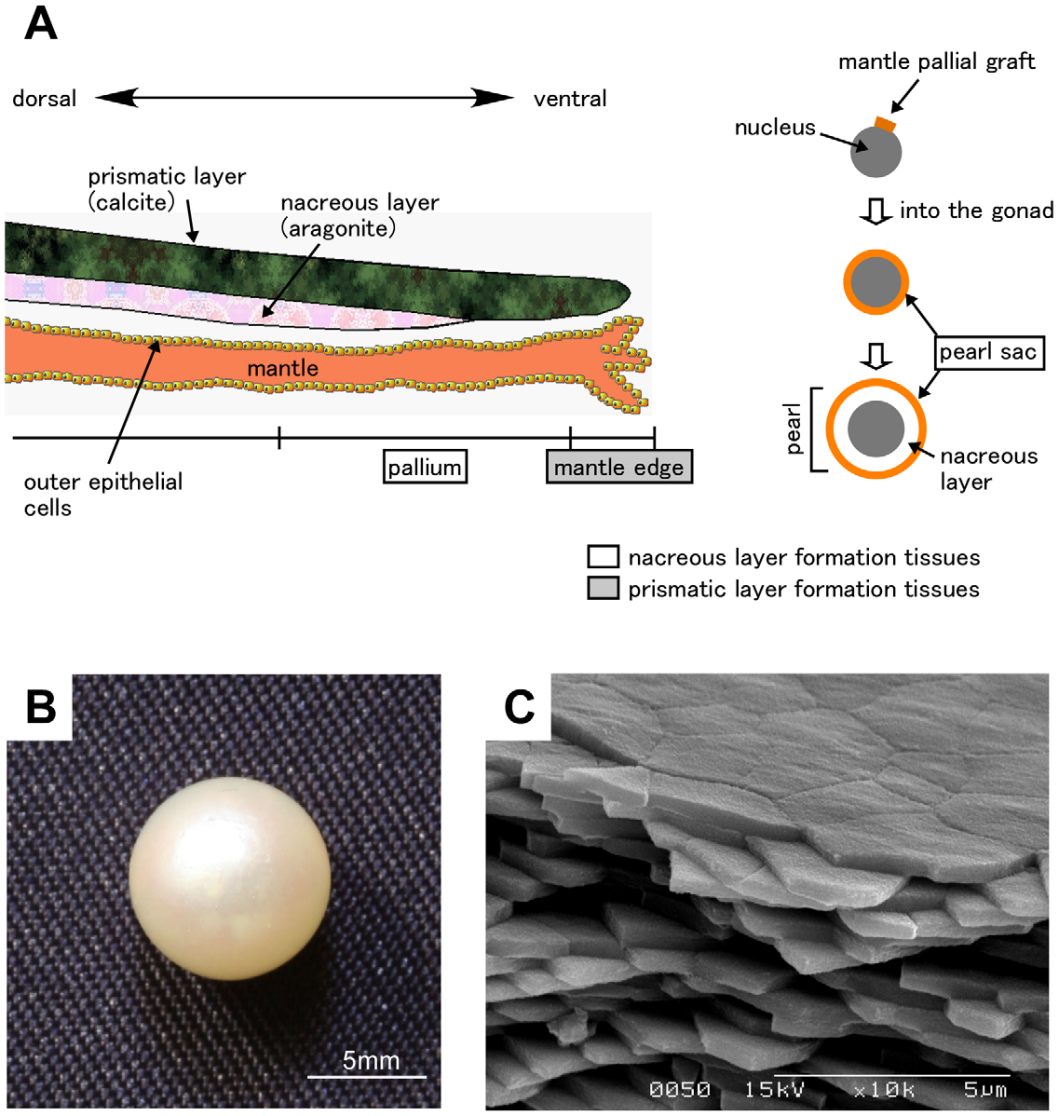
Tissues used for EST analysis. A, schematics of the shell and pearl sac of the Japanese pearl oyster *Pinctada fucata*. Two nacreous layer-producing tissues, pallium and pearl sac, and 1 prismatic layer-producing tissue, mantle edge, were used in this study. B, C, a pearl harvested from a pearl sac used in this study (B) and its peripheral microstructure revealed by scanning electron microscopy (C).

Because of its high industrial value, nacreous layer formation in the pearl oyster is a well-studied phenomenon. The shell of pearl oysters consists of 2 distinct structures: inner nacreous layers composed of aragonite and outer prismatic layers composed of calcite (reviewed in [1]). One of the most interesting questions in biomineralization is how 2 polymorphs of calcium carbonate are produced in the same organism. Pearl oysters initiate shell formation with amorphous calcium carbonate, which is transformed into either calcite or aragonite [2]–[4]. These transformation processes are thought to be regulated by proteins secreted from epithelial cells in outer mantle tissues [1], [5]. These proteins form a biomineral framework and regulate the nucleation and growth of calcium carbonate. The differences in the composition of proteins secreted from the outer mantle tissues generate the calcite and aragonite polymorphs of calcium carbonate [1], [6]. Nacreous and prismatic layers are formed in different regions of the outer mantle. The ventral part of the mantle (mantle edge) forms the prismatic layers, whereas the dorsal part of the mantle (pallium) forms the nacreous layers (Fig. 1A). In pearl oyster culture, grafts from recipient pallia are transplanted with nuclei into the gonad of mother oysters. Pearl sac tissues are formed by proliferation of epithelial cells originating from the outer mantle graft where various proteins are secreted to form the nacreous layers [7], [8] (see Fig. 1A). Extensive studies have been conducted to identify the proteins responsible for shell formation by screening proteins contained in the shell and genes specifically expressed in the mantle (reviewed in [1]). A wide variety of proteins and genes have been identified and their functions in shell formation have been partially characterized. So far, however, there have been no systematic studies on the entire transcriptome in pearl oyster shell formation and our understanding of the molecular mechanisms involved in pearl oyster shell formation is fragmented. Annotated gene sets for pearl oyster in the DDBJ/EMBL/GenBank databases are quite limited and there is no high-density whole-genome database.

Here, we present the first report of deep sequencing of ESTs from the pearl oyster *Pinctada fucata* using a next-generation sequencer. The aim of this study was to develop a high-throughput experimental approach for transcriptome analysis in shell-formation tissues including mantle edge, pallium, and pearl sac of *P. fucata.* We sequenced 260477 reads and identified 29682 unique sequences. We also screened novel shell formation-related gene candidates by a combined analysis of sequence and expression data sets.

## Materials and Methods

### RNA isolation and library construction

*P. fucata martensii* mantle and pearl sac tissues were collected in September 2009 from 4 individuals maintained at the Mikimoto pearl farm, Mie, Japan. Mantle pieces had been grafted to all individuals for pearling in April 2009. To address whether the pearl sac actually produced the nacreous layers, peal oysters were harvested and pearls in the peal sac were observed by scanning electron microscopy (Fig. 1B, C). The mantle edge and pallial mantle tissues were separated from the mantle and these tissues, including the pearl sac, were preserved in RNAlater (Applied Biosystems, Foster City, CA, USA).

Total RNA was extracted with the RNeasy Lipid Tissue Mini Kit (QIAGEN, Hilden, Germany) and 3′-fragment sequencing was performed at Operon Biotechnology, Tokyo, Japan, where we employed pyrosequencing to sequence the transcriptome, using the GS FLX 454 system (Roche, Basel, Switzerland). An important advantage of this platform is that we are able to conduct transciptome analysis even in organisms for which we have no genome or EST data sets. The preparation of 3′-fragment cDNAs was as follows: equal quantities of total RNAs from 4 individuals were pooled and fragmented by ultrasonication. Poly(A)^+^ RNAs were isolated from the fragmented total RNAs and a RNA adapter was ligated to the 5′-phosphate of the poly(A)^+^ RNA. First-strand cDNA synthesis was performed using an oligo(dT)-adapter primer. The 3′-fragment cDNA products were PCR-amplified to about 40 ng/µl.

### Sequence analysis and bioinformatics

3′-fragment sequences were obtained by the Genome Sequencer FLX protocol (Roche). After quality trimming of raw reads, a *de novo* assembly using MIRA assembler ver. 2.9.45×1 and the BLAST Clust program from NCBI were used to the assemble reads. Clustered EST data sets were annotated using blast2go (http://blast2go.bioinfo.cipf.es/). This program takes a query collection of nucleotide sequences and uses the blastx algorithm to search a UniProt database by gene ontology (GO). We used an expect value of 1E-10 for the blastx searches. When multiple annotations were returned, the annotation with the best score was saved. To search the functional domains of the novel proteins we identified, we used the InterProScan program (http://www.ebi.ac.uk/Tools/InterProScan/).

Known nacreous and prismatic gene sequences were searched using the local blastn and tblastn algorithms against the *P. fucata* EST data set: MSI60 [9], MSI25 (DDBJ/EMBL/GenBank accession number: AB210136), Pif177 [10], nacrein [11], N66 [12], *P. fucata* mantle gene 1 (PFMG1) [13], amorphous calcium carbonate-binding protein (ACCBP) [14], N16s [12], [15], calmodulin-like protein (CaLP) [16], N19 [17], aspein [18], prismalin-14 [19], Lys-rich matrix proteins 1-4 (KRMPs 1–4) [20], prismin [21], prisilkin-39 [22], and shematrins 1–7 [23]. Novel gene candidates with possible shell-formation associations were searched using the same method against EST data sets of abalone *Haliotis asinina* (8341 ESTs), Mediterranean mussel *Mytilus galloprovincialis* (19753 ESTs) and Japanese scallop *Mizuhopecten yessoensis* (9100 ESTs) in the DDBJ/EMBL/GenBank databases.

Gene expression was represented as transcripts per million (TPM) which corresponds to (total reads of a given gene/total reads in the tissue) ×10^6^. Cluster analysis based on the TPM was performed by CLUSTER3.0 (http://bonsai.hgc.jp/~mdehoon/software/cluster/software.htm#ctv) using Euclidean distance, and Java TreeView (http://jtreeview.sourceforge.net/) was used to visualize clustering relationships.

All sequence data sets obtained in this study have been registered to the DDBJ Sequence Read Archive (project number 63487).

### 5′Rapid amplification of cDNA ends (RACE)

We performed 5′-RACE to determine the full-length sequence of gene 000096. Gene-specific primers 000096-R1 (5′-GGTTTTGGTTTGACAGGTGC-3′) and 000096-R2 (5′-CGCCCAATTGCACGTTCCTC-3′) were designed from a partial gene sequence. Total RNA was extracted from the pallial mantle with the ISOGEN kit (Nippon Gene, Tokyo, Japan). 5′-RACE was performed with the GeneRacer Advanced RACE kit (Invitrogen, Carlsbad, CA, USA). 5′-RACE products were subcloned into pGEM-T (Promega, Madison, WI, USA) and sequenced on an ABI3100 Genetic Analyzer (Applied Biosystems).

## Results

### Library features

**Figure 2:**
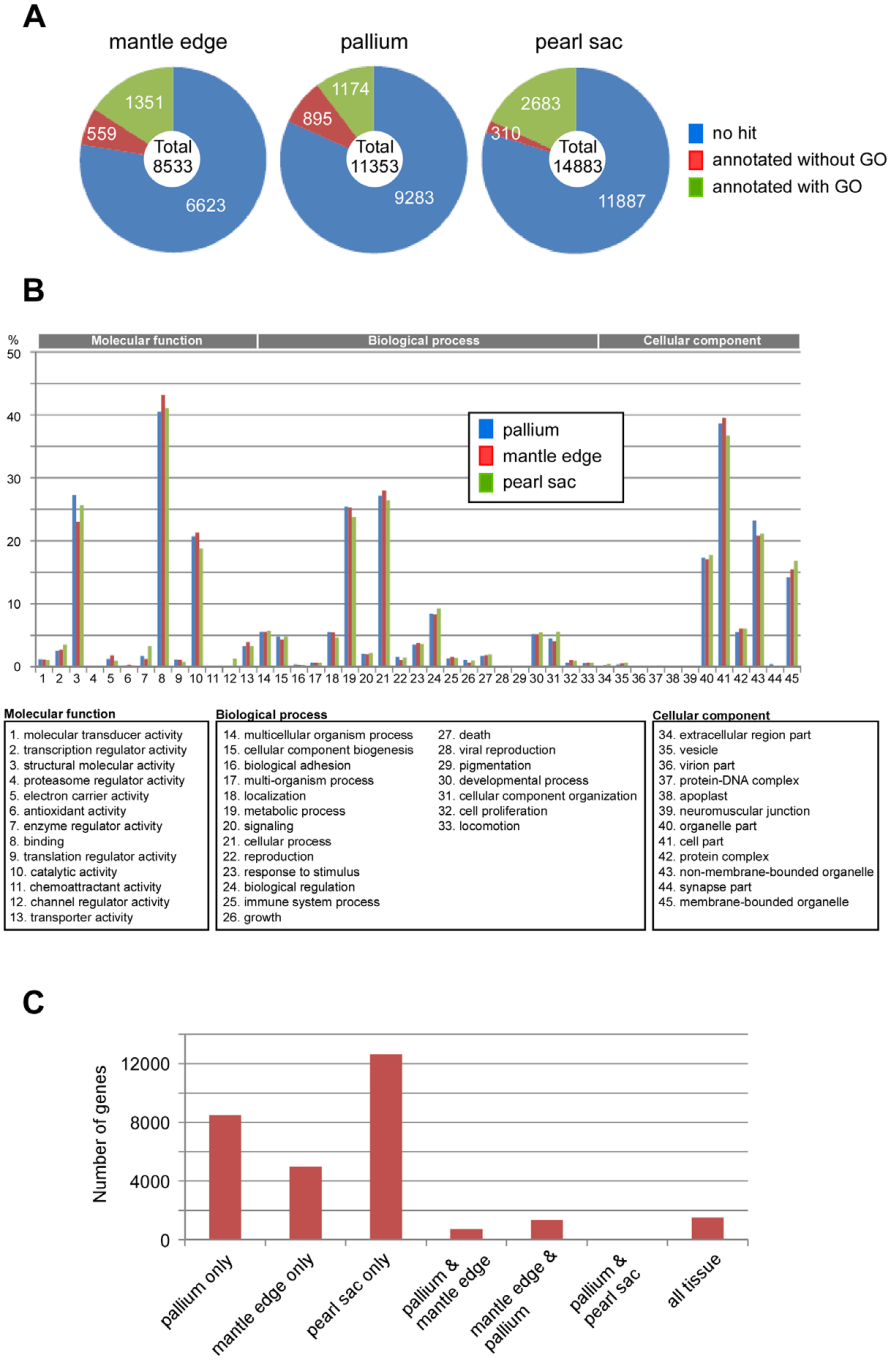
Composition and distribution of 29682 genes identified in this study. A, annotation of genes in each tissue. Blastx algorithm with cut-off E-value of 1.0E-10 was used for annotation. B, gene ontology of genes annotated with GO terms. C, distribution of genes in 3 tissues.

We acquired 508,730 reads (Table 1). After quality trimming, 260477 single pass 3′-reads, 68690 from the mantle edge, 83629 from the pallium, and 108095 from the pearl sac produced 29,682 unique sequences automatically numbered by the BLAST Clust program as 000001-029682. The average contig length was 495 bp (range 118–2065 bp) and the average total read was 8.8 (range 1–2460). Based on the blastx algorithm with cut-off at 1E-10, most of the 29682 genes shared no homology with known protein sequences (Fig. 2A). GO composition of annotated genes was almost the same in each tissue (Fig. 2B), whereas the expression of 29682 genes was variable between tissues (Fig. 2C).

**Table 1:** Summary of GS-FLX sequencing results.

Feature	Number
Read number	508730
-Mantle edge	147870
-Pallium	176175
-Pearl sac	184685
Number of assembled reads	260477
-Mantle edge	68690
-Pallium	83692
-Pearl sac	108095
Average read length (bp)	354
-Mantle edge	361
-Pallium	357
-Pearl sac	347
Number of contigs	29682
-Mantle edge	8533
-Pallium	11353
-Pearl sac	14883
Average length of contigs (bp)	495
-max.	2065
-min.	118
Average reads of contigs	8.8
-max.	2460
-min.	1

Because of the low accuracy of determining the expression levels of genes with low reads, we cut off genes under 40 reads in the following expression analyses. Our EST library contained 796 genes with ≥40 reads. Total reads from 796 genes accounted for 52% of the total transcriptome sampled in this study. The characteristics of genes with ≥40 reads are shown in Fig. S1.

### Genes with ≥40 reads predominantly expressed in nacre-producing tissues

First, we screened shell formation-related gene candidates by a simple strategy of retrieving genes by their dominant expression in nacre-producing tissues including the pallium and peal sac, both of which showed at least a 2-fold difference in expression in comparison to those in the mantle edge. Among the 796 genes with ≥40 reads, 29 genes were retrieved (Table 2). Two known *P. fucata* genes, MSI25 and N151 (DDBJ/EMBL/GenBank accession number: AB534773), were also retrieved. MSI25 is a nacreous gene and is predominantly expressed in the pallium. Although the function of N151 has not been characterized, our results strongly suggest its participation in nacreous layer formation. Two ribosomal protein genes, 000027 and 001194, were also identified by this screen. Furthermore, 4 genes, 000488, 000591, 001181, and 001311, shared significant homology with E-value under 1.0E-10. The remaining 21 genes showed no significant homology to known sequences.

**Table 2:** Genes dominantly expressed in nacreous layer-producing tissues.

	Gene		TPM		Total reads	Homology by Blastx	E-value	Source	Description
		ME	P	PS					
known *P. fucata* gene	N151(000362)	218	753	500	132	-	-	*Pinctada fucata*	-
	MSI25(000348)	29	406	120	49	-	-	*Pinctada fucata*	-
homology with known genes	000027	641	1744	3432	561	ribosomal protein l32	4.81E-55	*Sipunculus nudus*	structural constituent of ribosome
	000488	102	215	962	129	small nuclear ribonucleoprotein	1.08E-37	*Salmo salar*	RNA binding
	000591	44	203	777	104	zinc finger protein 593	3.70E-29	*Danio rerio*	transcription factor
	001311	87	335	241	60	elongation factor 2	3.09E-82	*Drosophila virili*	translation
	001181	44	108	435	59	dynein light chain	1.50E-45	*Brugia malayi*	cytoskeletal activity
	001194	58	179	194	40	60s ribosomal protein l37	3.55E-34	*Eurythoe complanata*	structural constituent of ribosome
no homology with known genes	000096	437	1446	2701	443	no hit	-	-	-
	000280	204	741	981	182	no hit	-	-	-
	000302*	73	1099	194	118	no hit	-	-	-
	000629	160	394	361	83	no hit	-	-	-
	000298*	73	633	213	81	no hit	-	-	-
	000569		167	481	70	no hit	-	-	-
	000677	102	275	342	67	no hit	-	-	-
	001201	29	323	333	65	no hit	-	-	-
	000738	29	60	500	61	no hit	-	-	-
	000585	58	179	361	58	no hit	-	-	-
	000560	73	335	213	56	no hit	-	-	-
	001159	44	143	352	53	no hit	-	-	-
	000545	102	227	213	49	no hit	-	-	-
	000464	102	191	241	49	no hit	-	-	-
	000424	73	167	241	45	no hit	-	-	-
	000858	87	239	167	44	no hit	-	-	-
	000753	58	227	176	42	no hit	-	-	-
	000837	58	143	231	41	no hit	-	-	-
	000653	29	72	305	41	no hit	-	-	-
	000964	58	155	222	41	no hit	-	-	-
	000795	73	155	204	40	no hit	-	-	-

Genes expressed over 2 times higher in the pallium or pearl sac than in the mantle edge in terms of TMP are listed.

*These genes were also retrieved in Fig. 9.

Abbreviations are: TPM, transcripts per million; ME, mantle edge; P, pallium; PS, pearl sac.

### The full-length cDNA sequence of gene 000096

**Figure 3:**
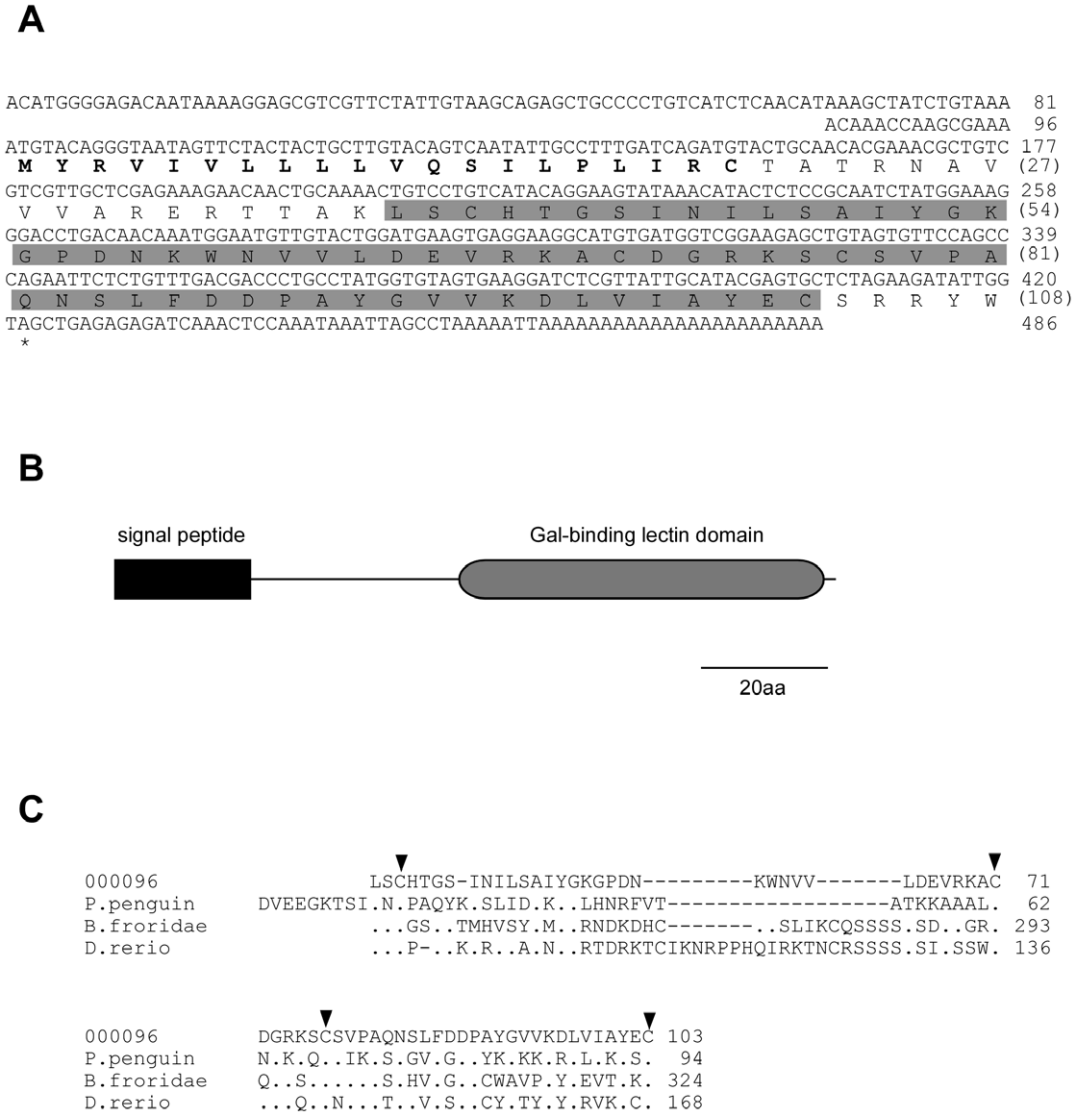
Molecular properties of 000096. A, complete cDNA and deduced amino acid sequences of 000096. Putative signal peptide and galactose-binding lectin domain are bold and shaded, respectively, in the amino acid sequences. B, motif structure of the protein encoded by 000096 containing a signal peptide and galactose (Gal)-binding lectin domain revealed by InterProScan. C, comparison of a putative Gal-binding lectin domain encoded by 000096 with those of lectins from pearl oyster *Pteria penguin* (DDBJ/EMBLE/GenBank accession number: AB037167.1), Florida lancelet *Branchiostoma floridae* (XP_002600399), and zebrafish *Danio rerio* (BX950205.10). Conserved cysteine residues are indicated by arrowheads.

Except for ribosomal proteins, the 443 total reads of 000096 exhibited the highest expression among genes expressed in nacre-producing tissues (Table 2). Interestingly, 000096 shared homology with a protein containing a galactose-binding lectin domain. Lectin is an important shell formation-related protein [24]–[27]. Therefore, we determined the full-length sequence of the 000096 cDNA by 5′-RACE (DDBJ/EMBL/GenBank accession number: AB635374). 000096 consisted of 486 bp encoding 108 amino acid residues (Fig. 3A). The InterProScan program demonstrated that 000096 contains an N-terminal signal peptide and a galactose-binding lectin domain at its C terminus (Fig. 3B). Figure 3C shows a comparison of the putative Gal-binding lectin domain encoded by 000096 with those of lectin domains from pearl oyster *Pteria penguin*, Florida lancelet *Branchiostoma floridae*, and zebrafish *Danio rerio*. The positions of cysteine residues in each domain were well conserved.

### Expression levels of known nacreous and prismatic genes in mantle edge, pallium and pearl sac

**Figure 4:**
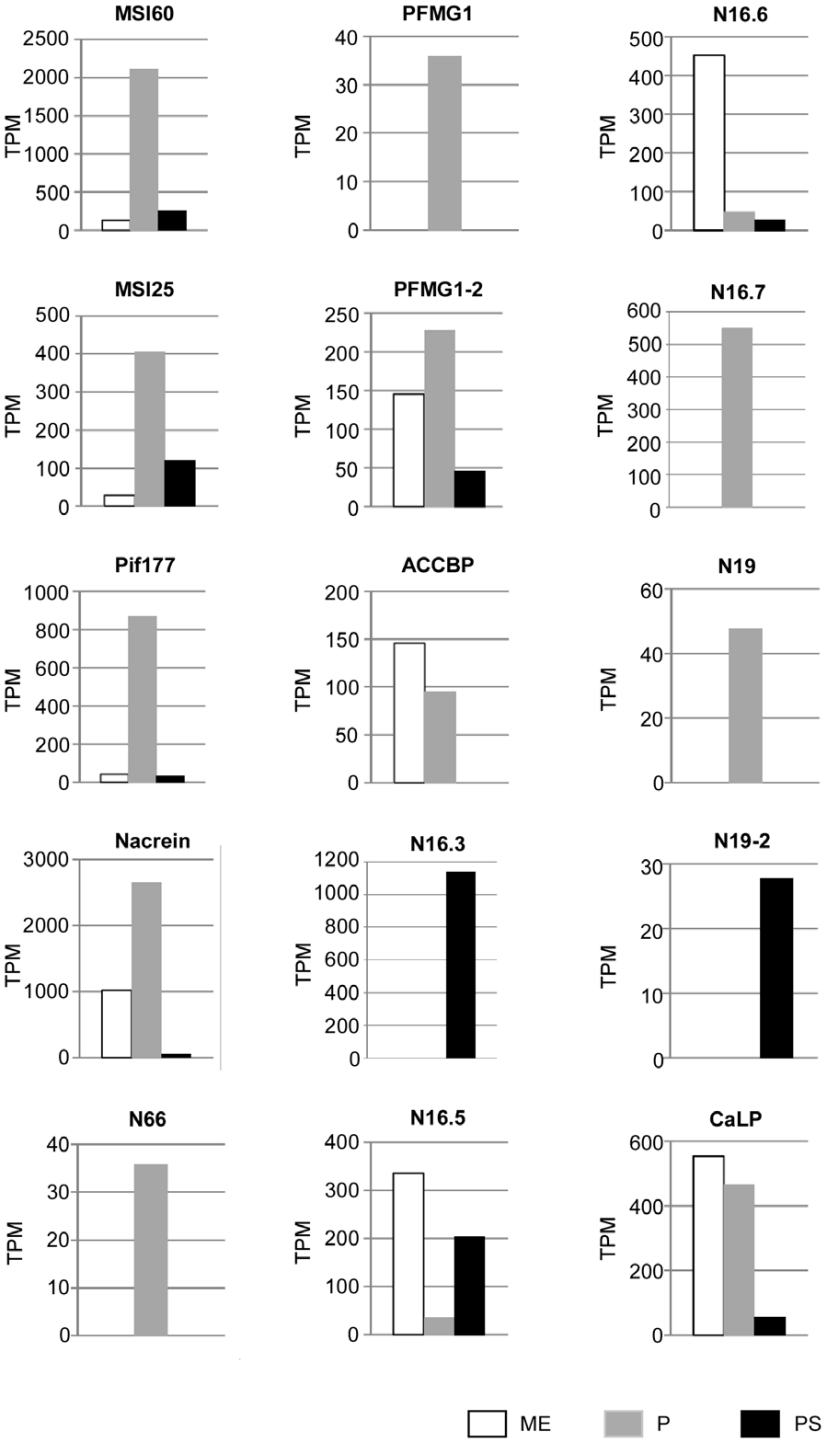
Expression of known nacreous genes. Abbreviations used are: PFMG1, *Pinctada fucata* mantle gene 1; ACCBP, amorphous calcium carbonate binding protein; CaLP, calmodulin-like protein; TPM, transcripts per million; ME, mantle edge; P, pallium; PS, pearl sac. PFMG1-2, N16.6, N16.7 and N19-2 are novel isoforms found in this study (Fig. S2).

**Figure 5:**
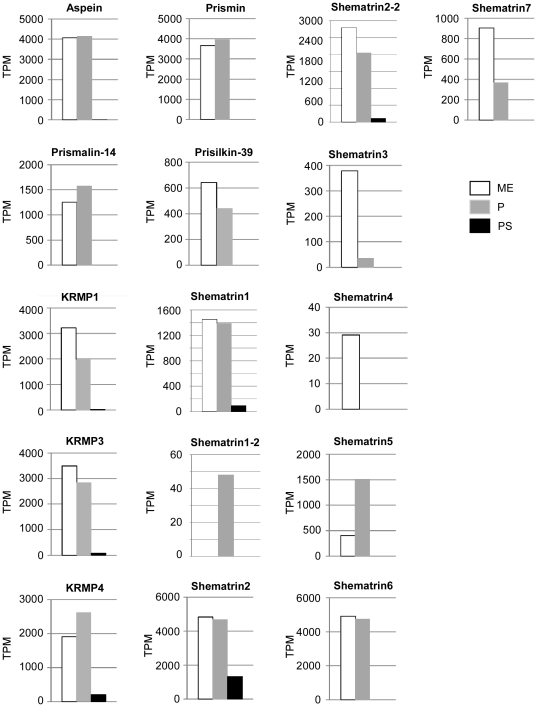
Expression of known prismatic genes. Abbreviations used are: KRMP, lysine-rich matrix protein; TPM, transcripts per million; ME, mantle edge; P, pallium; PS, pearl sac. Shematrin1-2 and shematrin2-2 are novel isoforms found in this study (Fig. S3).

We also examined the expression of known nacreous and prismatic genes in our EST libraries. Although many studies have identified genes and proteins that are related to nacreous and prismatic layer formation, functional analyses have been limited. Among the previously reported genes, we selected 11 nacreous and 14 prismatic genes from the pearl oyster *P. fucata* and related species *P. maxima* (Table S1). We detected novel isoforms of PFMG1 (PFMG1-2), N16s (N16.6 and N16.7), N19 (N19-2), shematrin1 (shematrin1-2) and shematrin2 (shematrin2-2) (Figs. S2 and S3); thus the expression of 31 genes was analyzed in the mantle edge, pallium, and pearl sac as shown in Figs. 4 and 5.

Eleven out of 15 previously reported nacreous genes showed predominant expression in the pallium, verifying their function in nacreous layer formation (Fig. 4, Table S1). Among the nacreous genes with ≥40 reads, MSI60, Pif177, nacrein and N16.7 showed the greatest expression in the pallium, suggesting their importance in nacreous layer formation. We noted that N66, PFMG1, N19 and N16.7 were specifically expressed in the pallium. In contrast, 4 nacreous genes (ACCBP, CaLP, N16.5 and N16.6) were more highly expressed in the mantle edge than in the pallium, suggesting their additional roles in prismatic layer formation. Although the expression of most nacreous genes was relatively low in the pearl sac, MSI60 and MSI25 were more highly expressed in this tissue than in the mantle edge. Interestingly, N19-2 and N16.3 were expressed specifically in the pearl sac.

Meanwhile, the expression of most known prismatic genes did not differ between the pallium and mantle edge except for shematrin3, shematrin4 and shematrin7, which were predominantly or exclusively expressed in the mantle edge. We noted that shematrin5 showed much greater expression in the pallium than in the mantle edge (Fig. 5, Table S1). Moreover, shematrin1-2 expression was specific to the pallium.

### Genes showing homology with known nacreous and prismatic ones

**Figure 6:**
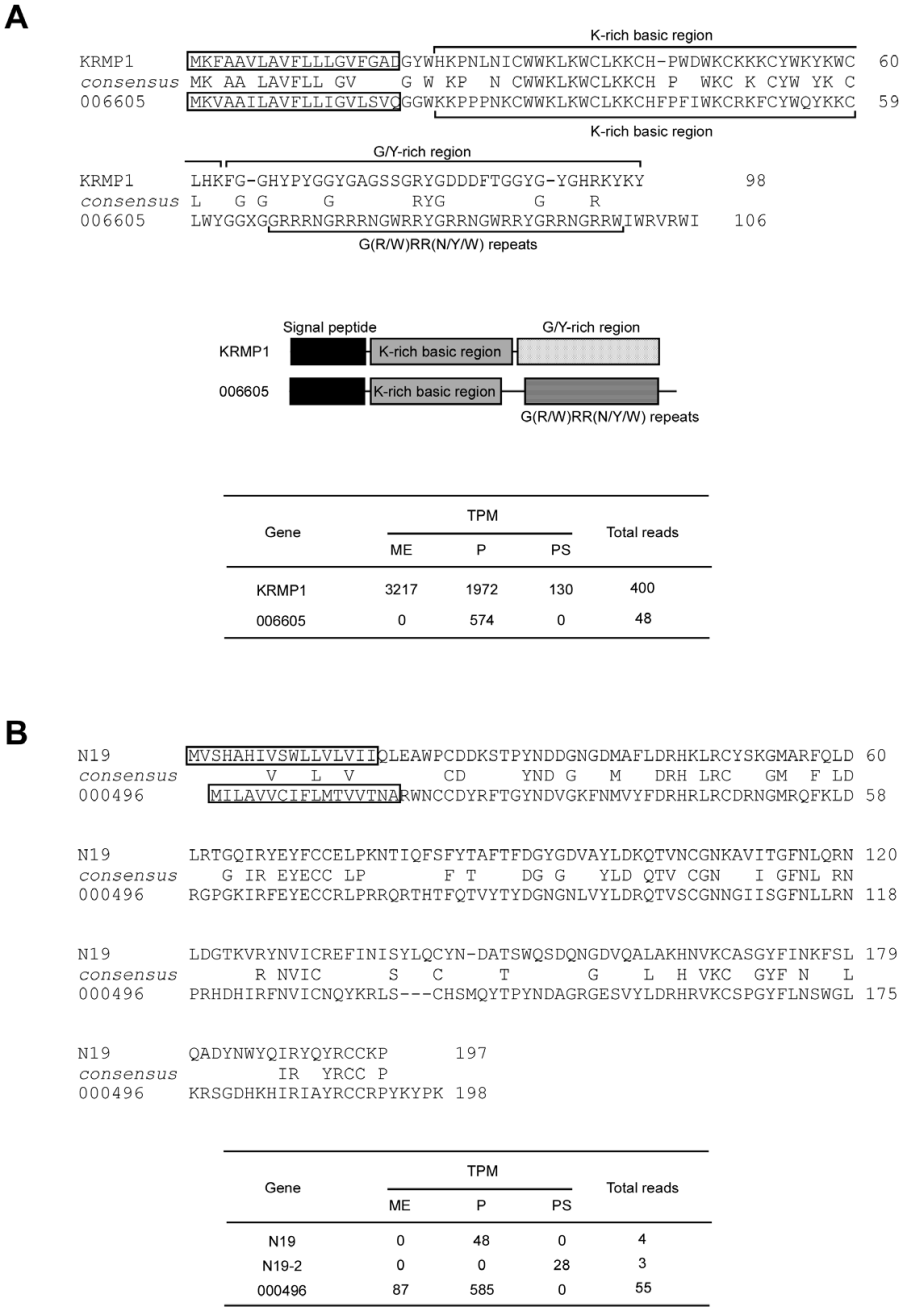
Shell formation-related gene candidates in *Pinctada fucata* EST library retrieved by their homology with KRMP1 and N19. A, homology of 006605 with lysine-rich matrix protein KRMP1 [20]. An N-terminal signal peptide is followed by a lysine-rich basic region and then by a glycine/tyrosine-rich domain (KRMP1) or G(R/W)RR(N/Y/W) repeats (006605) at the C terminus. Boxed sequences are putative signal peptides. The expression of 006605 is highly specific to the pallium. B, homology of 000496 with a nacreous gene, N19 [17]. The boxed sequences are putative signal peptides predicted by InterProScan. The expression of 000496 is highly specific to the pallium. An N19 isoform, N19-2, was found in this study (see Fig. 4 and Fig. S2). Abbreviations are: TPM, transcripts per million; ME, mantle edge; P, pallium; PS, pearl sac.

We screened the genes showing homology with known shell formation-related genes from 796 genes with ≥40 reads. The deduced amino acid sequences of genes 006605 and 000496 shared homology with KRMP1 and N19, respectively (Fig. 6). KRMP1 is a lysine-rich matrix protein expressed in mantle edge of *P. fucata* [20], consisting of an N terminal signal peptide, lysine-rich basic region, and a C terminal glycine/tyrosine-rich region (Fig. 6A). The protein encoded by 006605 also contained an N terminal signal peptide and lysine-rich basic region, but lacked a glycine/tyrosine-rich region. Instead of the glycine/tyrosine-rich region, the protein contained 7 G(R/W)RR(N/Y/W) repeats at its C terminus, suggesting that 006605 is functionally different from KRMP1.

N19 inhibits CaCO_3_ crystallization and is contained in the nacreous layer of *P. fucata* [17]. InterProScan indicated that N19 and the 000496 encoded protein both have N-terminal signal sequences (Fig. 6B), although the amino acid identity between these proteins is low (36%). The expression of 000496 and 006605 was highly specific to the pallium (Fig. 6), suggesting their role in the nacreous layer formation.

**Figure 7:**
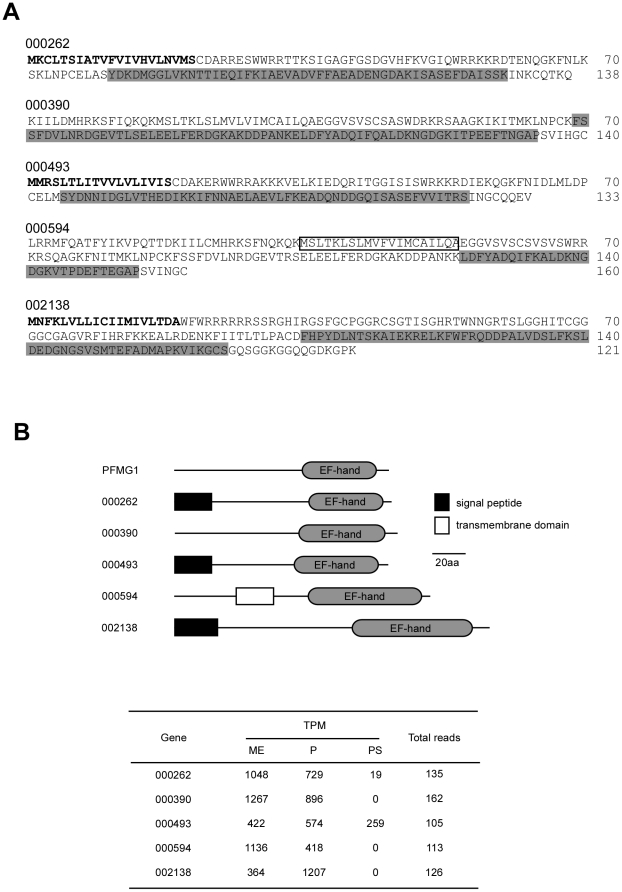
Shell formation-related gene candidates in *Pinctada fucata* EST library retrieved by homology with PFMG1. A, deduced amino acid sequences of 000262, 000390, 000493, 000594, and 002138. Putative signal peptides and EF-hand calcium-binding domains are in bold and shaded, respectively. A putative transmembrane domain in 000594 is boxed. B, structural properties of the genes and their expression patterns. All proteins contain EF-hand calcium-binding domains. Proteins encoded by 000296, 000493, and 0002138 contain N-terminal signal peptides, whereas 000594 has a transmembrane domain. The expression levels of 000262, 000390, and 000594 are higher in the mantle edge than in the pallium, whereas 000493 and 002138 are higher in the pallium than in the mantle edge. A PFMG1 isoform, PFMG1-2, was found in this study (Fig. 4 and Fig. S2). Abbreviations are: TPM, transcripts per million; ME, mantle edge; P, pallium; PS, pearl sac.

The PFMG1 sequence probe identified 5 genes from the EST library: 000262, 000390, 000493, 000594, and 002138 (Fig. 7A). Amino acid identities of these gene products with PFMG1 were 14–62%. We noted that 000390 and 000594 lacked an initial methionine and were thus considered to be larger molecules than those shown in Fig. 7A. PFMG1 was originally identified as one of the genes expressed in the mantle of *P. fucata* [13]. It encodes an EF-hand calcium-binding domain and promotes calcite formation *in vitro*. InterProScan indicated that all 5 proteins contain an EF-hand calcium-binding domain (Fig. 7B). The existence of this domain is thought to be the reason why these 5 genes were identified in the screen. However the structural properties of these genes are different from those of PFMG1. For example, 000262, 000493, and 002138 have signal peptides, whereas 000594 has a transmembrane domain (Fig. 7B), suggesting that these proteins are functionally different from PFMG1. Transcripts of 000262, 000390 and 000594 were detected in the mantle edge more than in the pallium, whereas the distribution of 000493 and 002138 transcripts was reversed, suggesting functional differences between these groups in shell formation.

Genes with homology to known nacreous and prismatic genes in Figs. 4 and 5 other than N19, KRMP1 and PFMG1 were not detected among the 796 genes with ≥40 reads.

### Screening of shell formation-related genes by cluster analysis for their expression patterns in different tissues

**Figure 8:**
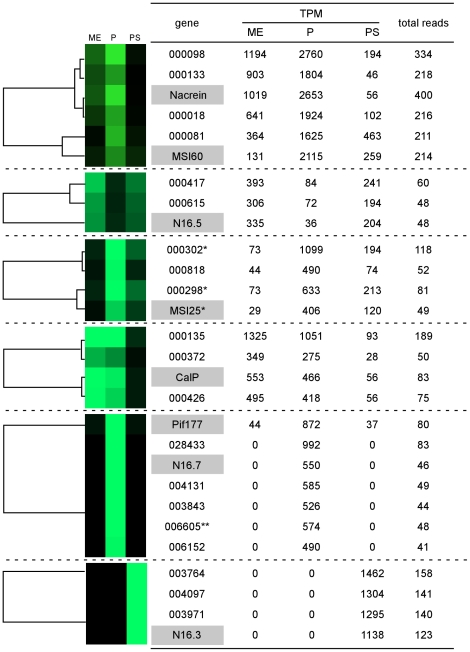
Genes clustered with known nacreous genes in their expression patterns in different tissues. The intensity of green color corresponds to the expression levels (TPM) of each gene in different tissues. Abbreviations are: ME, mantle edge; P, pallium; PS, pearl sac; TPM, transcripts per million. *These were also retrieved as genes specific to nacre-producing tissues in Table 2. **006605 shared homology with KRMP1 [20] (Table 3, Fig. 6A).

**Figure 9:**
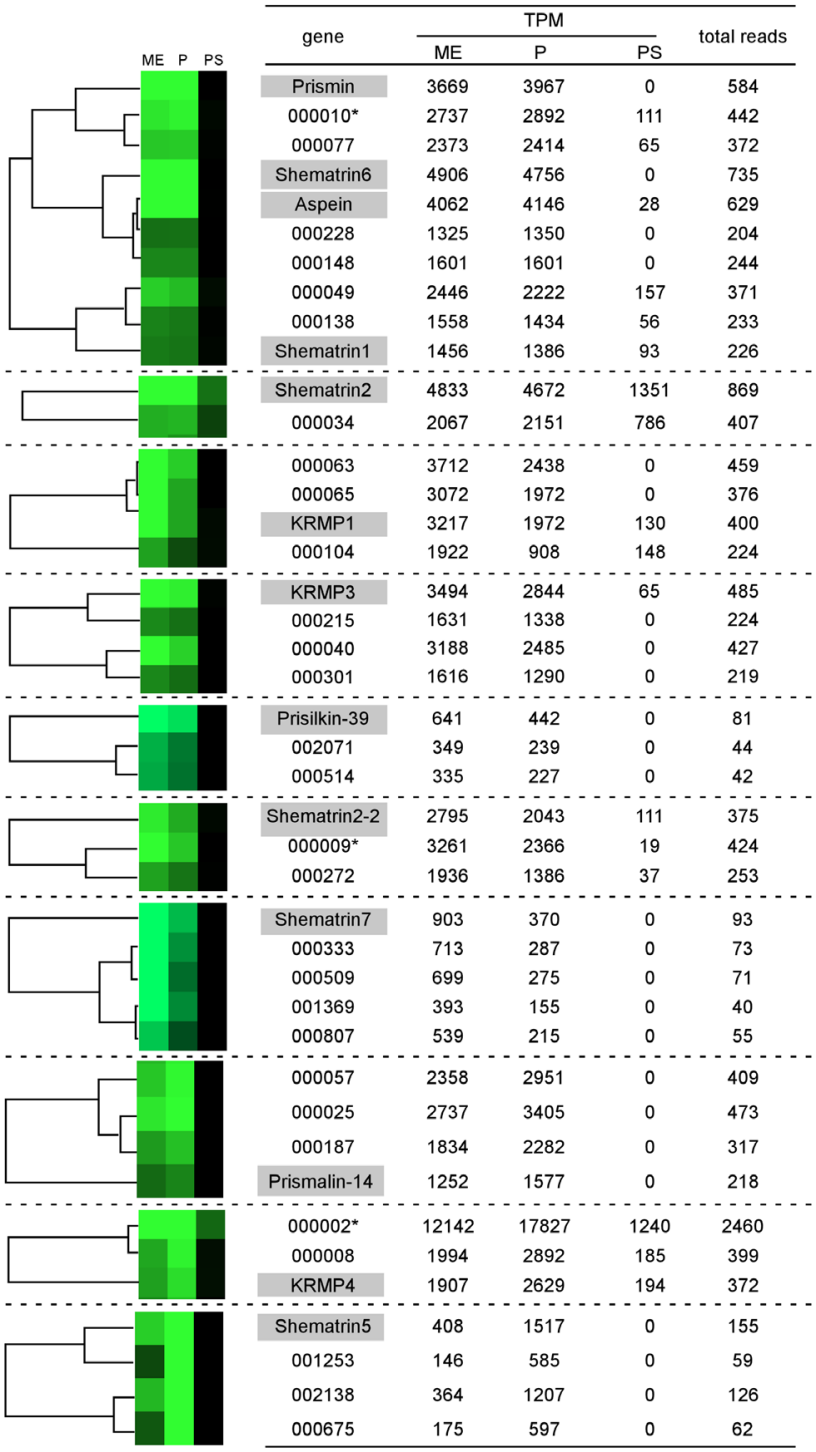
Genes clustered with known prismatic genes in their expression patterns in different tissues. The intensity of green color corresponds to the expression levels (TPM) of each gene in different tissues. *000010, 000002, and 000009 were identified as *Pinctada fucata* mantle gene 10 (PFMG10), mantle gene 4 (PFMG4), and mantle gene 5 (PFMG5), respectively (Table 3). Abbreviations used are: ME, mantle edge; P, pallium; PS, pearl sac; TPM, transcripts per million.

For further screening of novel shell formation-related gene candidates among the 796 genes with ≥40 reads, cluster analysis by CLUSTER3.0 was performed based on their expression patterns in different tissues (Fig. S4). We divided the genes into highly expressed (≥200 reads, 195 genes) and moderately expressed (40–199 reads, 601 genes) groups. Genes that clustered with known nacreous and prismatic genes were selected (Figs. 8 and 9). Note that N66, PFMG1, PFMG1-2, ACCBP, N16.6, N19, and N19-2 in Fig. 4, and shematrin1-2, 3, and 4 in Fig. 5 were excluded from this analysis because of their low expression levels (<40 reads, see Table S1).

**Table 3:** Candidates of the shell formation-related genes found by cluster analysis.

Gene	Homology by Blastx	E-value	Source	Description	Relation to the shell formation
**Clustered with known nacreous genes**					
*Clustered with nacrein & MSI60*					
*000098*	SCO-spondin	3.00E-13	*Bos taurus*	Secreted glycoprotein, Act in neuronal development	Unknown
000133	No hit	-	*-*	-	-
000118	No hit	-	*-*	-	-
000081	No hit	-	*-*	-	-
*Clustered with N16.5*					
*000417*	ATP synthase subunit B	2.00E-23	*Xenopus tropicalis*	ATP synthesis in mitochondria	Unknown
*000615*	Cytochrome b	1.00E-67	*Mytilus edulis*	Electron transport, ATP synthesis in mitochondria	Unknown
*Clustered with MSI25*					
000302	No hit	-	*-*	-	-
000818	No hit	-	*-*	-	-
000298	No hit	-	*-*	-	-
*Clustered with CalP*					
000135	No hit	-	-	-	
*000372*	Kunitz-like protease inhibitor	3.00E-27	*Ancylostoma caninum*	Serine protease inhibitor activity	Various protease inhibitors are contained in bivalve shells
000426	No hit	-	*-*	-	-
*Clustered with Pif177 & N16.7*					
028433	No hit	-	-	-	
*004131*	Predicted protein	5.00E-17	*Nematostella vectensis*	Sugar binding lectin domain	Lectin is contained in bivalve shells
003843	No hit	-	-	-	-
006605**	No hit	-	-	-	-
006152	No hit	-	-	-	-
*Clustered with N116.3*					
*003764*	Protein v1g120119	1.86E-23	*Nematostella vectensis*	Metalloprotease activity	Metaroproteases are important in the bone formation
004097	No hit	-	-	-	-
003971	No hit	-	-	-	-
**Clustered with known prismatic genes**					
*Clustered with prismalin-14*					
000057	No hit	-	*-*	-	-
000025	No hit	-	*-*	-	-
*000187*	Primary amine oxidase	5.00E-36	*Branchiostoma floridae*	Primary amine oxidase activity	Unknown
*Clustered with prismin, aspein, shematrin1, shematrin6*					
**000010** *	Mantle gene 10 (PFMG10)	6.00E-88	*Pinctada fucata*	Serine protease inhibitor domain	Expressed in mantle cells
000077	No hit	-	*-*	-	-
*000228*	Kazal-type serine proteinase inhibitor	4.00E-16	*Chlamys farreri*	Serine peotease inhibitor activity	Various protease inhibitors are contained in bivalve shells
000148	No hit	-	*-*	-	-
000049	No hit	-	*-*	-	-
000138	No hit	-	*-*	-	-
*Clustered with KRMP1*					
000063	No hit	-	*-*	-	-
000065	No hit	-	*-*	-	-
*000104*	Intermediate filament protein	3.50E-11	*Aplysia californica*	Cytoskeleton	Unknown
*Clustered with KRMP3*					
*000215*	Hypothetical protein	2.37E-19	*Hydra magnipapillata*	Unknown function	Unknown
000040	No hit	-	*-*	-	-
000301	No hit	-	*-*	-	-
*Clustered with KRMP4*					
**000002** *	Mantle gene 4 (PFMG4)	4.03E-72	*Pinctada fucata*	Unknown function	Expressed in mantle cells
000008	No hit	-	*-*	-	-
*Clustered with prisilkin-39*					
002071	No hit	-	*-*	-	-
000514	No hit	-	*-*	-	-
*Clustered with shematrin2*					
000034	No hit	-	*-*	-	-
*Clustered with shematrin2-2*					
**000009** *	Mantle gene 5 (PFMG5)	1.46E-40	*Pinctada fucata*	Unknown function	Expressed in mantle cells
000272	No hit	-	*-*	-	-
*Clustered with shematrin5*					
001253	No hit	-	*-*	-	-
002138**	No hit	-	*-*	-	-
000675	No hit	-	*-*	-	-
*Clustered with shematrin7*					
000333	No hit	-	*-*	-	-
000509	No hit	-	*-*	-	-
001369	No hit	-	*-*	-	-
*000807*	Protein W01F3.3a	1.00E-11	*Caenorhabditis elegans*	Kunitz-type serine protease inhibitor domain	Various protease inhibitors are contained in bivalve shells

*000002, 000009, and 000010 were identified as PFMG4, PFMG5, and PFMG10, respectively.

**006605 and 002138 shared homology with KRMP1 and PFMG1, respectively.

Genes identified as known *P. fucata* genes and sharing significant homology with known genes are boldfaced and italicized, respectively.

Among the known nacreous genes, nacrein and MSI60 formed a cluster with 4 genes (Fig. 8, Table 3). One of them, gene 000098, showed homology with bovine SCO-spondin with moderate significance (Table 3). N16.5 was expressed more abundantly in the mantle edge than in the pallium. N16.5 formed a cluster with another 2 genes with high homology to ATP synthase subunit B and cytochrome b (Table 3). MSI25 formed a cluster with 3 genes. MSI25, 00032, and 000298 were also identified as genes that are abundantly expressed in nacre-producing tissues (Table 1, see also Fig. 4). CaLP was expressed more abundantly in the mantle edge than in the pallium. CaLP formed a cluster with another 3 genes. One of these, 000372, encoded a protein with highly significant homology to a Kunitz-like protease inhibitor (DDBJ/EMBL/GenBank accession number: AF533590) (Table 3). The expression of Pif177 and N16.7 was highly specific to the pallium (see also Fig. 4) and clustered with 5 genes. One of them, 004131, shared homology with a predicted protein from *Nematostella vectensis* (DS469772) that contains a sugar-binding lectin domain. We noted that 006605 was also retrieved as a gene with homology to KRMP1 (Fig. 6B). N16.3 was abundantly expressed in and highly specific to the pearl sac (see Fig. 4). Three genes, 003764, 004097, and 003971, formed a cluster with N16.3 and showed similar expression patterns. Gene 003764 shared highly significant homology with a metalloendopeptidase (DS469673) (Table 3).

Among the prismatic genes, prismin, aspein, shematrin1, and shematrin6 were expressed both in the pallium and mantle edge at similar levels, and clustered with another 6 genes (Fig. 9, Table 3, see also Fig. 5). Among them, 000010 was identified as *P. fucata* mantle gene 10 (PFMG10) (Table 3). PFMG10 was originally isolated from a mantle cDNA library of *P. fucata* [13], and encodes a Kazal-type serine protease inhibitor with a follistatin-like domain. We noted that 000228 in the same cluster also shared a significant homology with a Kazal-type serine protease inhibitor from *Chlamys farreri* (EU183309) (Table 3). Shematrin2 was expressed in the mantle edge and pallium at a similar level, forming a cluster with one gene. KRMP1, KRMP3, prisilkin-39, shematrin2-2, and shematrin7 were expressed in the mantle edge more than in the pallium (Fig. 5). They formed clusters with another 3, 3, 2, 2 and 4 genes, respectively. Gene 000009 clustered with shematrin2-2 and was identified as *P. fucata* mantle gene 5 (PFMG5). PFMG5 was isolated from the mantle cDNA library of *P. fucata* [13], although its function has not been clarified. It was noted that 000807 clustered with shematrin7 and shared significant homology with a *Caenorhabditis elegans* protein containing a Kunitz-type serine protease inhibitor domain (CAX65076.1). Prismalin-14, KRMP4 and shematrin5, which were more highly expressed in the pallium than in the mantle edge, were clustered with another 3, 2, and 3 genes, respectively. Gene 002138 showed homology with PFMG1, which contains an EF-hand calcium-binding domain (Fig. 7). Gene 000002 was identified as *P. fucata* mantle gene 4 (PFMG4). PFMG4 in the mantle of *P. fucata* contains a complement component C1q domain [13].

### Distribution of shell formation-related gene candidates in other shellfish species

In this study, 79 shell formation-related gene candidates were retrieved from *P. fucata* EST data sets. We examined the presence of these candidates in other shellfish species including abalone *H. asinina*, Mediterranean mussel *M. galloprovincialis* and Japanese scallop *M. yessoensis*. Eighteen of the 79 genes were detected with significant homology in these species (Table 4), and 61 genes were unique to *P. fucata* and shared no similarity with ESTs in the comparator species.

**Table 4:** Distribution of shell formation-related gene candidates in different shellfish species.

*Pinctada fucata*		*Haliotis asinina*(8341 ESTs)			*Mytilus galloprovincialis*(19753 ESTs)			*Mizuhopecten yessoensis*(9100 ESTs)		
Gene	Description	Accession number	E-value	Mantle expression	Accession number	E-value	Mantle expression	Accession number	E-value	Mantle expression
000027	P & PS specific	GT277644.1	4.00E-69	yes	FL593283.1	9.00E-74	unknown	GR867732.1	3.00E-70	yes
000488	P & PS specific	no hit	-	-	no hit	-	-	GT569198.1	1.00E-39	unknown
000591	P & PS specific	no hit	-	-	FL490187.1	8.00E-42	unknown	GH735420.1	4.00E-58	unknown
001181	P & PS specific	GT273372.1	1.00E-58	yes	EH663240.1	5.00E-57	unknown	no hit	-	-
001194	P & PS specific	GT272497.1	4.00E-42	yes	no hit	-	-	GT568920.1	2.00E-39	unknown
001311	P & PS specific	no hit	-	-	FL497401.1	3.00E-99	unknown	no hit	-	-
000098	Clustered with nacrein & MSI60	no hit	-	-	FL497315.1	3.00E-23	unknown	no hit	-	-
000417	Clustered with N16.5	DW986478.1	5.00E-24	yes	FL594996.1	4.00E-33	unknown	no hit	-	-
000615	Clustered with N16.5	DY402875.1	2.00E-57	yes	FL593400.1	8.00E-18	unknown	FY301239.1	3.00E-47	unknown
000372	Clustered with CalP	GT274134.1	3.00E-13	yes	no hit	-	-	no hit	-	-
000426	Clustered with CalP	GT271665.1	2.00E-17	yes	AJ625332.1	1.00E-29	unknown	no hit	-	-
004131	Clustered with Pif177 & N16.7	no hit	-	-	AJ624088.1	1.00E-20	unknown	no hit	-	-
028433	Clustered with Pif177 & N16.7	no hit	-	-	FL492714.1	3.00E-12	unknown	no hit	-	-
000228	Clustered with prismin, aspein, shematrin1, shematrin6	no hit	-	-	FL499052.1	1.00E-15	unknown	no hit	-	-
000215	Clustered with KRMP3	no hit	-	-	FL500067.1	1.00E-18	unknown	no hit	-	-
000002	Clustered with KRMP4	no hit	-	-	FL490015.1	4.00E-17	unknown	no hit	-	-
000807	Clustered with shematrin7	GT274423.1	1.00E-15	yes	no hit	-	-	no hit	-	-
000496	N19-like sequence	no hit	-	-	FL498326.1	2.00E-25	unknown	no hit	-	-
000027	P & PS specific	GT277644.1	4.00E-69	yes	FL593283.1	9.00E-74	unknown	GR867732.1	3.00E-70	yes
000488	P & PS specific	no hit	-	-	no hit	-	-	GT569198.1	1.00E-39	unknown
000591	P & PS specific	no hit	-	-	FL490187.1	8.00E-42	unknown	GH735420.1	4.00E-58	unknown

Abbreviations are: P, pallium; PS, pearl sac.

## Discussion

Despite its economic importance, we do not have a comprehensive understanding of the molecular mechanisms involved in biomineralization in pearl oysters, where 2 distinct calcium carbonate crystals are formed in a precisely controlled manner. In this study, we constructed comprehensive expressed gene profiles in the shell-forming tissues of the pearl oyster *P. fucata*, and obtained 29682 unique sequences by using a next-generation sequencer (Table 1 and Fig. 2). Prior to this study, there were only several hundred mRNAs registered in the DDBJ/EMBL/GenBank databases for pearl oysters; we have now identified over 29000 novel gene sequences from *P. fucata*.

We also screened novel nacreous and prismatic gene candidates by a combined analysis of sequence and expression data sets. We successfully retrieved various novel candidate genes including those encoding protease, protease inhibitors, lysine-rich matrix protein, and secreting calcium-binding proteins. We also examined the transcription levels of known nacreous and prismatic genes in our EST library. Our data showed that known prismatic genes tended to show higher expression levels (2–1034 reads) than nacreous genes (3–400 reads) (see Figs. 4, 5, Table S1), which is consistent with the fact that shell formation is more strongly activated in the ventral region than in the dorsal region of the mantle [28].

In our EST libraries, most known nacreous genes showed higher expression levels in the pallium than in the mantle edge (Fig. 4), whereas most of the known prismatic genes were expressed in both mantle tissues and sometimes at higher levels in the pallium than in the mantle edge (see Fig. 5). Takeuchi and Endo [29] compared the expression levels of shell matrix protein genes between the pallium and mantle edge by real-time PCR and found that nacreous genes including MSI60, N16, and nacrein were specifically or dominantly expressed in the pallium, whereas prismatic genes including aspein, MSI31 [9], and prismalin-14 were expressed in the mantle edge and pallium at similar levels. These results are somewhat consistent with the data in our study. They separated the pallium into dorsal, middle, and ventral parts (Fig. 1A), and found that prismatic genes were expressed in the ventral part of the pallium [29]. The likely reason why various known prismatic genes were expressed in the pallium in this study is that we did not separate the pallium into dorsal and ventral parts. Alternatively, these prismatic genes may also play an important role in nacreous layer formation. Jackson et al. [30] posited that prismatic genes such as shematrins and KRMPs are also important components of nacreous layer formation in *P. maxima*.

It was also notable that our EST data sets contained genes encoding isoforms of PFMG1, N19, N16s, shematrin1, and shematrin2. Interestingly, tissue-specific expression was clearly observed in each isoform of N19, N16, and shematrin1 (Figs. 4 and 5). N19 was detected only in the pallium whereas N19-2 was detected only in the pearl sac. The expression of N16.6, N16.7, and N16.3 was highly specific to the mantle edge, pallium, and pearl sac, respectively, whereas N16.5 was expressed in both mantle edge and pearl sac. It has been reported that N19 and N16s are contained in the nacreous layer of *P. fucata* and inhibit calcium carbonate crystallization [12], [17], [18]. Gene duplication often causes divergent expression of paralogous genes [31]. The relationships between these functional differences in tissue-specific isoform expression are under investigation in our research group.

Although the pallium and pearl sac have the same function in terms of nacre-producing activity, our data indicates that the expression patterns of various nacreous genes are not consistent between the 2 tissues. On the other hand, Inoue *et al*. [32], [33] reported that expression patterns of several shell formation-related genes were similar in the pearl sac and pallium, but different from those in the mantle edge. One possibility is that contaminating tissues surrounding the pearl sac decreased the expression of shell formation-related genes in our pearl sac preparation. The pearl sac is composed of very thin tissues covering the pearl layer; thus, surrounding tissues may contaminate the sample. The other possibility is that different cellular environments may affect expression levels. Molluscan shell formation employs extracellular calcification processes [34]: epithelial cells in the outer mantle secrete organic compounds (extrapallial fluid) and then calcification proceeds in the fluid-filled space (extrapallial space). In the pearl sac, the extrapallial space is supposed to be comparatively small [34]. Therefore, the low expression of certain genes may be sufficient for calcification in the pearl sac.

Calcium ion is an important component of shell formation. However, the molecular mechanisms of molluscan calcium homeostasis including recruitment of calcium ion to the extrapallial fluids remain unclear. *P. fucata* has at least 3 calcium-binding proteins in the mantle. CaLP [17] and PFMG1 [13] were cloned from a mantle cDNA library and found to induce nucleation of the nacreous layer. Huang *et al*. [35] isolated an EF-hand calcium-binding protein, EFCBP, from *P. fucata* and showed that its expression was immediately upregulated when the shell was damaged. We identified 5 novel genes which encode proteins with EF-hand calcium-binding domains (Fig. 7B). Tissue-specific expression patterns suggest that proteins 000493 and 0002138 function in nacreous layer formation, whereas proteins 000262, 000390 and 000594 function in prismatic layer formation.

We also identified a novel gene 000096 which encoded a protein with a putative signal peptide and galactose-binding lectin domain (Fig. 3). Glycoprotein and polysaccharide are major organic components of the molluscan shell and thus lectins, carbohydrate-binding proteins, are likely key regulatory factors in shell formation. To date, at least 4 molluscan shell proteins including mucoperlin [36], dermatropin [37], calprismin [38] and nacrein [39] have been shown to be glycosylated. Various lectins have been isolated from marine bivalves including *P. fucata* [24], [40], [41]. Perlucin, a C-type lectin, was isolated from the nacreous layer of the abalone inner shell and proved to promote calcium carbonate crystallization [25]–[27]. These lines of evidence together with the tissue-specific expression pattern of 000096 strongly suggest its role in nacreous layer formation.

Bedouet *et al*. [42] reported the existence of protease inhibitors in the nacreous layer of the pearl oyster *P. margaritifera*. We also identified a gene encoding a protease inhibitor, SPI, by screening a suppression-subtractive hybridization (SSH) cDNA library of *P. fucata* [43]. Lie *et al*. [13] reported a putative secreting protein, PFMG12, from the ESTs of mantle cells of *P. fucata*, demonstrating its sequence homology with a protease inhibitor containing a Kunitz-like domain. Abalones *Haliotis rufescens* and *H. laevigata* contain nacreous proteins including lustrinA [44] and perlwapin [45], respectively, exhibiting sequence homology with a protease inhibitor. Furthermore, Kunitz-type and Kazal-type serine protease inhibitors were recently reported in a proteomic analysis of freshwater bivalve nacre [46]. In our EST library, 2 novel genes, 000372 and 000228, shared significant homology with Kunitz-like and Kazal-type protease inhibitors, and were clustered with known shell formation-related genes (Table 3). These lines of evidence, together with our findings, suggest that protease inhibitors may have a key function in shell formation.

Proteases are important regulators in bio-calcification. In human beings, bone formation is a dynamically steady state balanced by osteoblasts and osteoclasts [47]. Various proteases function to digest extracellular matrix proteins for bone resorption [48]. For example, a metalloprotease mediates collagen digestion. Gene 003764 was retrieved by its homologous expression with N16.3 (Fig. 8, Table 3) and shared significant homology with a metalloendopeptidase (see Table 3). The functional role of 003764 in shell formation is an interesting question to be addressed.

KRMP is a matrix protein containing lysine-rich basic and glycine/tyrosine-rich domains [20]. The lysine-rich domain interacts with negatively charged ions such as those in bicarbonate or acidic matrix proteins [20], whereas the glycine/tyrosine-rich domain is thought to function in protein polymerization via cross-linking. KRMPs are thought to work as linker proteins through these domains between acid-soluble proteins and hydrophobic framework proteins in *P. fucata* prismatic layer formation [20]. Gene 006605 lacked the glycine/tyrosine-rich domain but retained the lysine-rich domain (Fig. 6A). Instead of the glycine/tyrosine-rich domain, 006605 contained 7 tandem repeats of G(R/W)RR(N/Y/W) at its C terminus. Tandem repeats are often found in framework proteins contained in various biominerals including molluscan shells [1]. Furthermore, 006605 clustered with known nacreous genes such as Pif177 and N16.7, which are specifically expressed in the pallium (Figs. 4 and 8). These results strongly suggest that 006605 is a novel framework protein in *P. fucata* nacreous layer formation.

In our cluster analysis based on expression data by 454 sequencing, nacreous and prismatic genes formed well-defined clusters (Figs. 4, 5, 8 and 9), indicating that genes with similar functions in terms of nacreous and prismatic layer formation were successfully clustered. Therefore, novel genes clustered with known nacreous and prismatic genes are likely to function in the corresponding layer formations. Genes 002138 and 006605 exhibited sequence homology with known shell formation-related gene, PFMG1 and KRMP1, respectively (Figs. 6 and 7), and formed groups with known nacreous and prismatic genes (Figs. 8 and 9). Therefore, these seem to be good candidates as novel shell formation-related genes. In addition, we found genes 000002 (PFMG4), 000009 (PFMG5), and 000010 (PFMG10) with high expression levels in the mantle edge and pallium were clustered with known prismatic genes (Fig. 9, Table 3). Although PFMG4, PFMG5 and PFMG10 were originally isolated from a mantle cDNA library of *P. fucata* [13], their functions have remained unclear. Our results strongly suggest their roles in prismatic and nacreous layer formation.

We retrieved 79 shell formation-related gene candidates from *P. fucata*. Most of the genes, however, shared no similarity with sequences in the EST data sets from other shellfish species (Table 4). Conversely, well-known shell formation-related genes such as AP7 (DDBJ/EMBL/GenBank accession number: AF225916) and perlinhibin (Swiss-Prot accession number: P85035) from abalone were not detected in our *P. fucata* EST data sets (data not shown). Jackson *et al*. [30] also showed large-scale compositional differences in mantle EST data sets between pearl oyster *P. maxima* and abalone *H. asiana*. These results suggest that highly varied gene sets are involved in shell formation in each species.

In conclusion, we constructed EST data sets from the nacre- and prism-producing tissues in the pearl oyster *P. fucata* and found 29682 unique sequences containing novel gene candidates for nacreous layer formation, although further functional analyses *in vitro* and *in vivo* are required to characterize their activities and functions. In this study, we focused on the major expressed genes (51% of the transcriptome extracted in this study). However, these results do not necessarily mean that other minor genes are not important in shell formation. Microarray analysis blotting of all 29682 genes will be a powerful tool to provide better understanding of the molecular mechanisms involved in molluscan biomineralization.

## Supporting Information

Figure S1 **Composition of genes with ≥40 reads (A) and gene ontology of genes annotated with GO terms (B).**
(TIF)

Figure S2 **Comparison of the amino acid sequences of isoforms of PFMG1 (A), N16 (B) and N19 (C).** Residues that differ from the top sequence are indicated in bold letters. N-terminal sites of PFMG1-2 and N19-2 were not determined due to lack of their 5′-sequecenes. N16.1, N16.2, N16.3 and N16.5 were already registered in the DDBJ/EMBL/GenBank databases [15], [17]. We found 2 additional isoforms named N16.6 and N16.7 in our EST database.
(TIF)

Figure S3 **Comparison of the amino acid sequences of the shematrin1 (A) and shematrin2 (B) isoforms.** Residues that differ from the top sequence are indicated in bold letters. N-terminal sites of shematrin1-2 and shematrin2-2 were not determined due to lack of 5′-sequecenes.
(TIF)

Figure S4 **Cluster analysis of genes with ≥200 reads (A) and 40–199 reads (B) based on their expression patterns in different tissues.** The intensity of green color corresponds to the expression level (TPM) of each gene in different tissues. Known nacreous and prismatic genes are indicated by arrows. Abbreviations are: ME, mantle edge; P, pallium; PS, pearl sac.
(TIF)

Table S1 Known nacreous and prismatic genes and their expression levels.
(XLSX)
