# Supplementary figures and images for: Deep Sequencing of ESTs from Nacreous and Prismatic Layer Producing Tissues and a Screen for Novel Shell Formation-Related Genes in the Pearl Oyster
Source: PLoS One. 2011 Jun 22;6(6):e21238. doi: 10.1371/journal.pone.0021238 (PMC3120837; doi:10.1371/journal.pone.0021238)

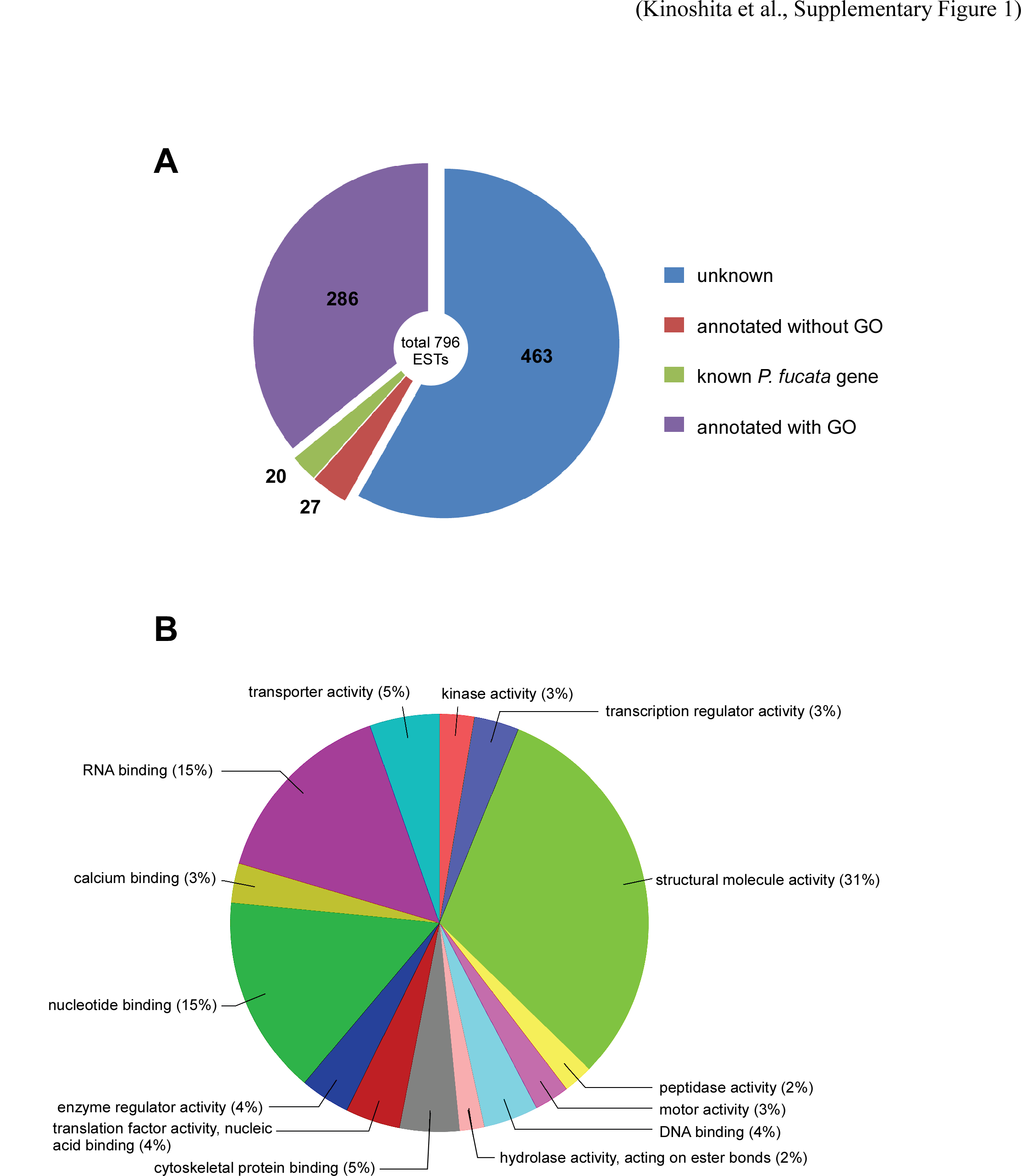

Supplement: Figure S1 — Composition of genes with ≥40 reads (A) and gene ontology of genes annotated with GO terms (B). (TIF) [file pone.0021238.s001.tif]

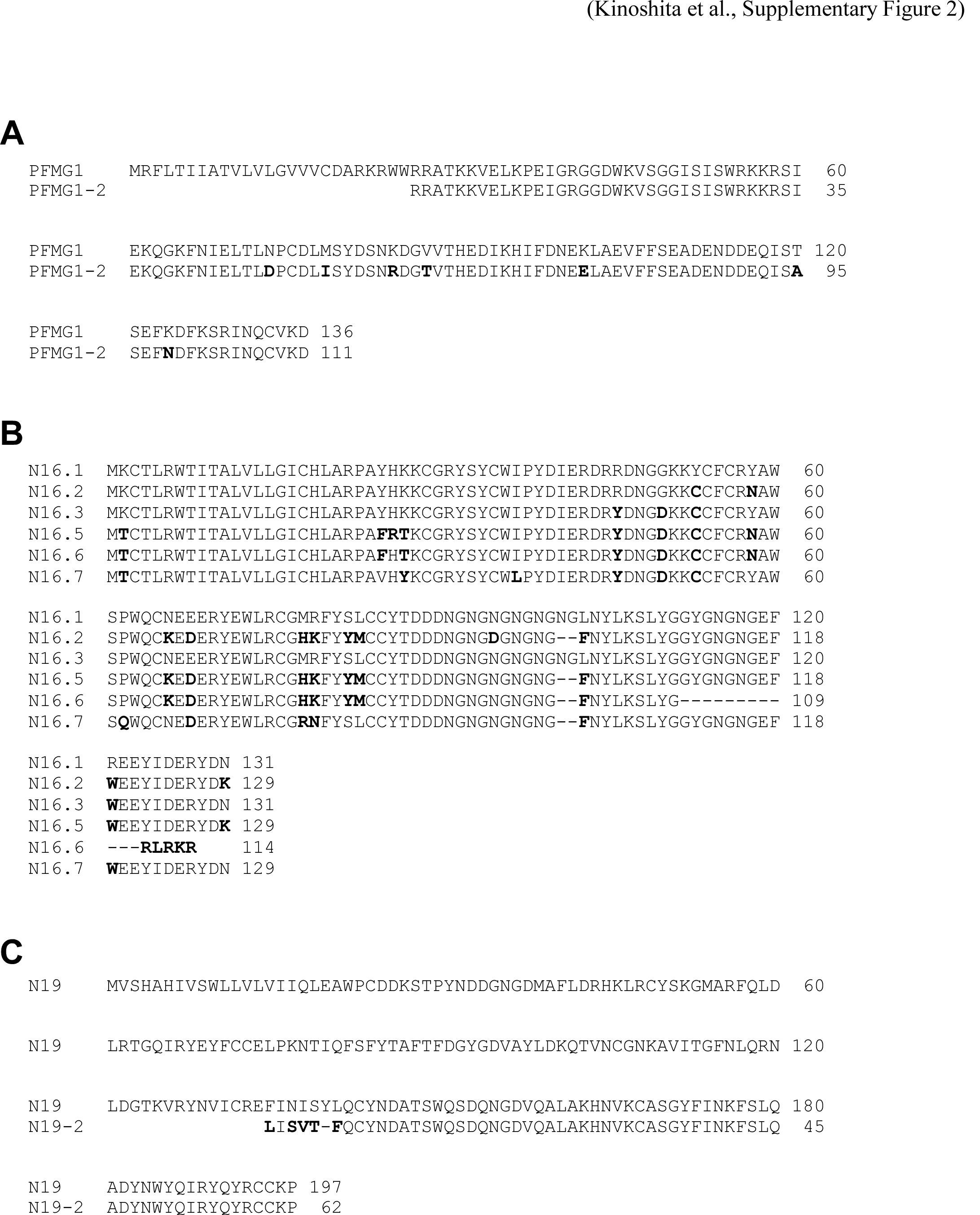

Supplement: Figure S2 — Comparison of the amino acid sequences of isoforms of PFMG1 (A), N16 (B) and N19 (C). Residues that differ from the top sequence are indicated in bold letters. N-terminal sites of PFMG1-2 and N19-2 were not determined due to lack of their 5′-sequecenes. N16.1, N16.2, N16.3 and N16.5 were already registered in the DDBJ/EMBL/GenBank databases [15], [17]. We found 2 additional isoforms named N16.6 and N16.7 in our EST database. (TIF) [file pone.0021238.s002.tif]

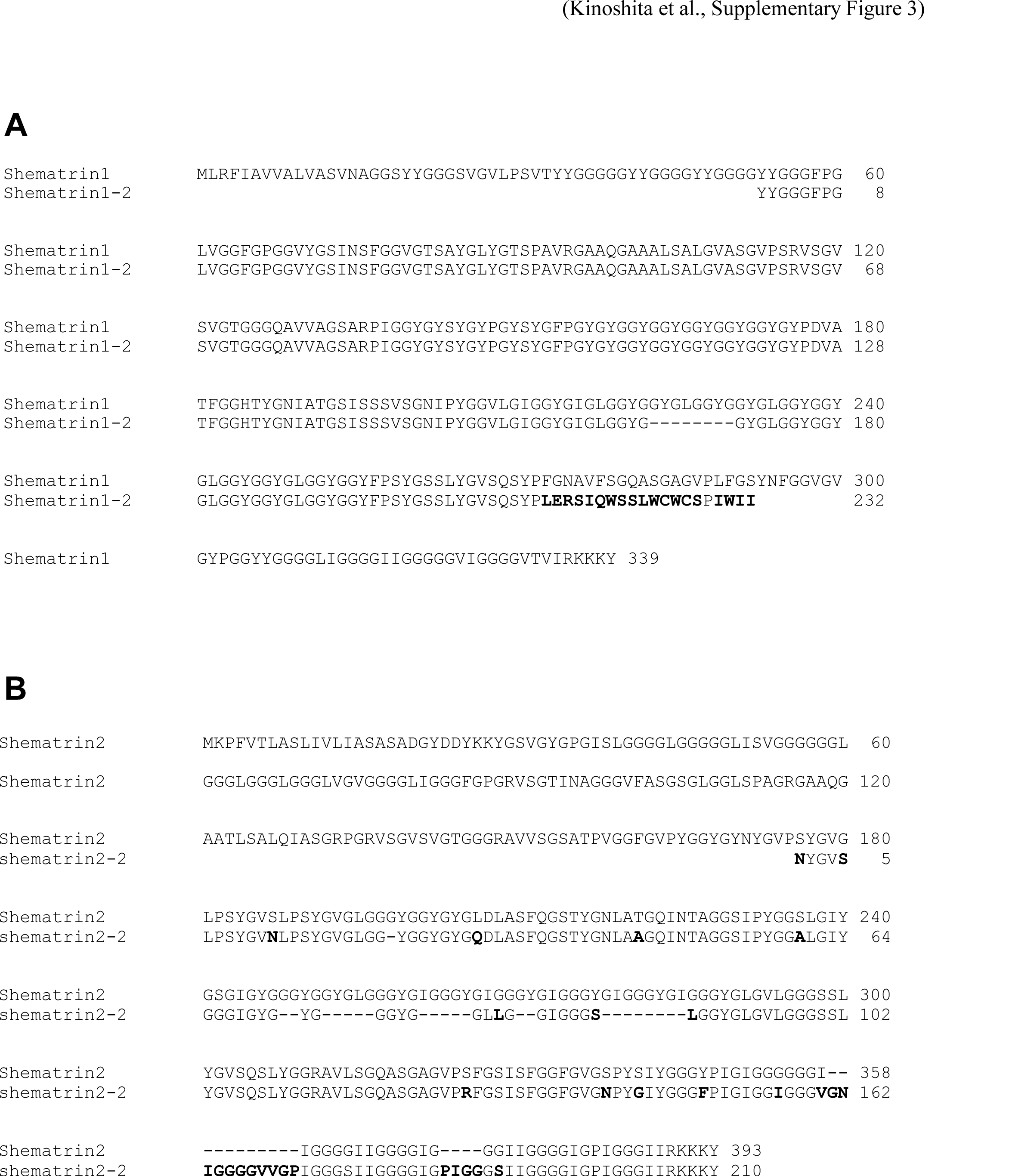

Supplement: Figure S3 — Comparison of the amino acid sequences of the shematrin1 (A) and shematrin2 (B) isoforms. Residues that differ from the top sequence are indicated in bold letters. N-terminal sites of shematrin1-2 and shematrin2-2 were not determined due to lack of 5′-sequecenes. (TIF) [file pone.0021238.s003.tif]

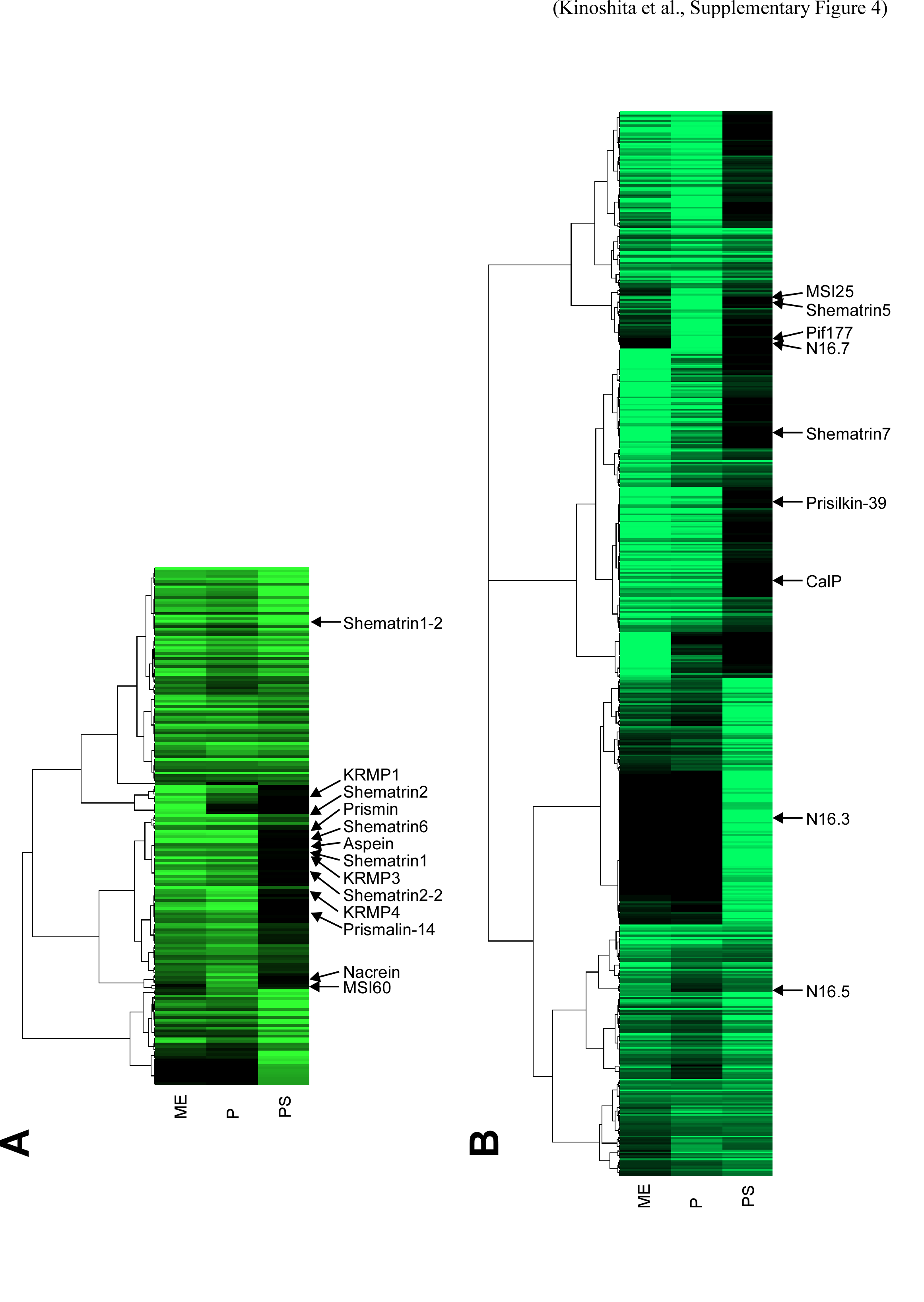

Supplement: Figure S4 — Cluster analysis of genes with ≥200 reads (A) and 40–199 reads (B) based on their expression patterns in different tissues. The intensity of green color corresponds to the expression level (TPM) of each gene in different tissues. Known nacreous and prismatic genes are indicated by arrows. Abbreviations are: ME, mantle edge; P, pallium; PS, pearl sac. (TIF) [file pone.0021238.s004.tif]
